# Eligibility of Medical Students to Serve as Principal Investigator: An Evidence-based Approach

**DOI:** 10.7759/cureus.7025

**Published:** 2020-02-18

**Authors:** Mohammad H Rajab, Abdalla M Gazal, Muhammad Alkawi, Khulood Kuhail, Fouad Jabri, Faizah A Alshehri

**Affiliations:** 1 Epidemiology and Public Health, Alfaisal University, Riyadh, SAU; 2 Internal Medicine, Alfaisal University, Riyadh, SAU; 3 Neurology, King Abdulaziz City for Science and Technology, Riyadh, SAU; 4 Biostatistics, Epidemiology and Public Health, College of Medicine, Alfaisal University, Riyadh, SAU; 5 Biomedical Sciences and Bioethics, Alfaisal University, Riyadh, SAU

**Keywords:** medical students, evidence-based approach, research involving human subjects, principal investigator, alfaisal university

## Abstract

Determining the eligibility of principal investigators (PIs) is a challenging task, especially at an academic institution. The prevailing practice within the academic community is not to grant PI status to students. There is a lack of studies that have investigated students’ eligibility to serve as PI. This study aimed to explore the faculty and students’ perceptions of the PI eligibility of medical students. A secondary objective was to assist the university in developing an evidence-based PI eligibility policy. To achieve the study aims, the investigators developed and validated a survey that targeted both faculty and students of Alfaisal University, College of Medicine (COM). In total, 53 faculty (four were administrators), and 135 medical students responded. The response rates were approximately 50% and 12% for faculty and students, respectively. Of the faculty, 62% reported that medical students are not PI eligible without the supervision of a faculty member. Of the students, 77% reported that they were not qualified to serve as PI. The results of the current study support the practice at most universities of not allowing medical students to serve as PI.

## Introduction

Research remains an essential pillar of most academic institutions despite the encountered challenges [1]. One of the challenges is determining the eligibility of a student to serve as a principal investigator (PI) on a study involving human subjects [2]. Another challenge is the need for more physicians to design and direct clinical research projects [3].

The PI is responsible for the overall administrative, fiscal, scientific, and ethical aspects of a research project and supervises research staff and students. Besides, the PI adheres to the institutional policies, and the institutional review board (IRB) policies and procedures regarding the safety and protection of human subjects, and good clinical practice guidelines [4,5]. In Saudi Arabia, there is also a need for assuring compliance with the National Committee of Bioethics (NCBE) regulations [6]. Granting that the PI may delegate tasks to members of the research team, the PI is eventually responsible for all aspects of the study.

Universities usually develop their PI eligibility policies and procedures. Exceptions to such policies are made on a case-by-case basis [7]. Universities require a specific educational background and research experience for someone to serve as PI. Unfortunately, many universities do not offer the required training and mentorship, and thus, most PIs learn on the job [3]. As a result, there has been a recent interest in the medical literature to evaluate the PI's duties and responsibilities [8]. Such evaluation helps in the planning for proper training and in providing adequate support systems [8].

Most universities do not consider students, including medical students, as PI eligible. However, few universities allow a student to serve as PI if accompanied by a qualified faculty member who has oversight responsibility. Faculty supervision ensures compliance with the IRB directives and the university policies and procedures [7].

Serving as PI on a human subject research project is a prestigious goal for young clinicians and medical students [8]. According to the American National Institutes of Health, human subject research is research conducted by an investigator (whether a professional or a student) on living individuals that obtain information through intervention or interaction with the individuals [9]. However, not all the research done at academic institutions meets the definition of human subject research. For example, classroom projects, where data are not intended for use outside the classroom, do not meet the definition of human subject research [10].

While there are no universal guidelines that prevent medical students from serving as PI, students’ eligibility for PI status is restricted. For example, Oregon State University disqualifies not only graduate and undergraduate students but also graduate assistants, postdoctoral fellows, and clinical fellows from serving as PI [11]. At Stanford University, eligibility to act as PI is a privilege limited to members of the Academic Council and the faculty [12]. 

With some exceptions, defining the PI eligibility criteria is usually based on who may receive funds on behalf of the university [7]. Occasionally, a sponsor may request that a student be listed as PI. In this case, the institution requires that a qualified faculty has oversight responsibility for the proposed PI to ensure the scientific and ethical conduct of research [10,13]. 

The Massachusetts Institute of Technology (MIT) allows students to serve as PI on exempt studies (for a thesis or dissertation) [14]. Exempt status is defined as minimal risk research that does not include the collection of identifiable information or the use of drugs or devices. Hence, students may act as PI at MIT; however, a faculty member must be recruited as a sponsor who has oversight responsibility for the student [14]. Likewise, Harvard University permits students to serve as PI if an eligible faculty member is also listed on the IRB application [15,16].

In Saudi Arabia, King Saud University has a program called “Undergraduate Research Support.” Such a program allows undergraduate students to act as a PI with the supervision of a faculty member [17]. Besides, the NCBE guidelines do not exclude students from the role of PI. An individual who wishes to act as PI on a study involving human subjects must receive basic training in bioethics and subsequently register as an investigator on the NCBE website [6].

In sum, the predominant practice at most academic institutions is not to allow medical students to serve as PI. Similarly, Alfaisal university does not allow medical students to serve as PI without the supervision of a qualified faculty member. Over the years, many medical students have complained against the institutional PI eligibility policy [2]. The argument was that the institution uses a non-evidence-based method to select PIs for research projects that are designed to produce evidence. In other words, the institution needs to support its policy on PI eligibility with evidence. Unfortunately, there is a paucity of studies that have investigated medical students' eligibility. This study aimed to explore the faculty and students’ perceptions on the eligibility of medical students to serve as PI. A secondary objective is to assist the university in developing an evidence-based PI eligibility policy.

## Materials and methods

We conducted a cross-sectional survey to solicit the feedback of faculty and students of the Alfaisal University, College of Medicine (COM). Located in Riyadh, Saudi Arabia, Alfaisal is a private, nonprofit, research-based, recently established university with over 3,000 students representing four colleges and over 40 nations [18]. The university’s IRB approved the study. The study investigators developed a self-administered online survey using Google Forms®. The survey contained an introductory paragraph that informs participants of the study’s aims, the confidentiality of their responses, and the freedom to withdraw at any point during the study period.

The survey contained a combination of closed- and open-ended questions. The closed-ended questions included inquiries into participants’ demographics, research experience, and medical students’ eligibility to act as a PI of studies involving human subjects. Open-ended questions included solicitation of recommendations to help develop the institutional PI eligibility guidelines. In addition, the survey included two parts; one was intended for faculty and administrators, and the other was intended for medical students.

As a part of the survey validation process, eleven COM faculty and students were asked to pilot-test the survey, out of which ten completed the survey. The pilot-test revealed, among other things, that it takes approximately 5-10 minutes to complete it. Eventually, several questions were modified or removed.

To start the study, we sent an introductory email to members of the target population. The target population consisted of faculty, administrators, and students (years 1 - 5) of the COM. The survey informed the target population of the study’s aims and solicited their participation. Next, the survey’s web-link was sent to the members of the target population using the institutional email system. Subsequently, two follow-up email reminders were emailed to the same groups. Graduate students and faculty and students who participated in the pilot-test of the study survey were excluded.

Data were automatically entered into an electronic database by the survey company Google Forms®. The data analysis involved two steps. The first used descriptive statistics by way of contingency tables. Next, specific relationships between categorical variables were examined using the Pearson χ2 test or Fisher’s Exact test, as appropriate. Data management and analysis were carried out using the Statistical Package for the Social Sciences (SPSS Inc., Chicago, IL), version 21 for windows. A significance level of p < 0.05 was used.

## Results

The study survey targeted both faculty and students of the COM. In total, 53 faculty (49 faculty and four administrators) and 135 medical students responded. The responses of the four administrators were included in the faculty category since most of the targeted administrators are full-time faculty at the COM. The response rates for faculty and medical students were approximately 50% and 12%, respectively. Of the faculty, 59% were females, 64% were Bachelor of Medicine, Bachelor of Surgery (MBBS) or Medical Doctor (MD) holders, and 36% were Doctor of Philosophy (PhD) or Master of Science (MS) holders. The baseline characteristics of faculty survey respondents are depicted in Table 1.

**Table 1:** Baseline characteristics of faculty survey respondents (N=53)

	%
Gender	
Male	41
Female	59
Degree	
Bachelor of Medicine, Bachelor of Surgery (MBBS), or Medical Doctor (MD)	64
Doctor of Philosophy (PhD) or Master of Science (MS)	36
Position	
Faculty	93
Administrator	7

The faculty who participated in the survey were asked if medical students are PI eligible for studies involving human subjects without faculty supervision. Of respondents, 62% reported that medical students are not PI eligible without the supervision of faculty advisers. Sub-group analysis revealed that physician faculty were more supportive of student’s PI eligibility than other faculty members (84 vs. 50, p < 0.05), respectively. The COM faculty responses by degree are summarized in Table 2.

**Table 2:** Faculty opinions regarding the eligibility of medical students to serve as principal investigator without faculty supervision by degree (N=53)

Degree	Eligible N (%)	Not eligible N (%)
Bachelor of Medicine, Bachelor of Surgery (MBBS) or Medical Doctor (MD)	17 (50)	17 (50)
Doctor of Philosophy (PhD) or Master of Science (MS)	3 (16)	16 (84)
Total	20 (38)	33 (62)

Of the medical student respondents, 52% were females. All medical students had high school certificates as their highest degree. The baseline characteristics of medical students’ survey respondents are presented in Table 3.

**Table 3:** Baseline characteristics of medical students’ survey respondents (N=135)

	%
Gender	
Male	48
Female	52
Degree	
High school certificate	100

We also collected data on medical student’s research education, research training, and research participation using a check-all-that-apply question. Of respondents, 75% reported having enough knowledge and training to conduct research, 46% were able to interpret research study findings, and only 19% knew how to implement research findings into medical practice. In terms of participation in studies involving human subjects, 59% of respondents have not participated, 28% served as investigators, 11% participated as study subjects, and only 2% served as a PI. Twenty-four percent reported receiving formal training in research ethics (e.g., local or international online course, or symposium). Research-related activities of medical students are presented in Table 4.

**Table 4:** Research-related activities of medical students (N=135) ^*Check all that apply question^

	%
Enough education and training to*	
Conduct research	75
Interpret research findings	46
Implement research findings into practice	19
Participation in human subject research	
None	59
As principal investigator	2
As investigator	28
As participant	11
Formal training in research ethics	
Yes	42

Of the medical student survey respondents, 49% were aware of the responsibilities of a PI for a study involving human subjects, and 62% were interested in serving as a PI. Only 23% reported being qualified to serve in the role of a PI. Responses of medical students regarding awareness for, interest in, qualification for serving as a PI for a study involving human subjects are summarized in Table 5.

**Table 5:** Medical students’ awareness of and interest in serving as principal investigator of human subject research (N=135)

	%
Aware of the roles and responsibilities of principal investigator	49
Interested in serving as principal investigator*	62
Qualified to serve as principal investigator*	23

The study team also solicited faculty and students’ feedback regarding student’s PI eligibility. Most survey respondents reported that students are not eligible to act as a PI on studies involving human subjects. Overall, the stated reasons were similar for both groups. Reasons for students not being eligible included a lack of knowledge and training to conduct quality research, being too busy with academic work to take on such a time-demanding task. In addition, few faculty members commented that allowing students to serve as PI for human subject research may negatively impact the reputation of the institution.

Other survey respondents strongly believed that medial students are PI eligible. They reported that PI experience is needed to elevate the levels of knowledge and research experience of medical students. Besides, they emphasized that a compromise must be made to establish a middle ground, i.e., students should be permitted to act as a PI if they meet specific criteria that would be placed by the IRB and the institution. Notably, the NCBE regulations do not exclude students from serving as a PI. Survey respondents' reasons for and against the eligibility of students to serve as PI are summarized in Table 6.

**Table 6:** Summary of feedback about the eligibility of medical students to serve as principal investigator

Not Eligible	Eligible
Too busy to take on such a demanding task	Interest in serving as principal investigator
Must be supervised by a qualified faculty	To minimize the potential for being taken advantage of
Do not have enough knowledge to conduct quality research	The support of Institutional Review Board
Requires formal training and specific qualifications	To prepare medical students for leadership
Do not have enough expertise in biomedical ethics	The regulations of the Saudi National Committee of Bioethics do not disqualify students from serving as principal investigator
A lack of maturity	Faculty may not have enough protected time to serve as principal investigator
Allowing students to serve as principal investigator may negatively impact the reputation of the institution	Medical students take courses in biostatistics, epidemiology, and evidence-based medicine and are encouraged to conduct research
	Clerkship students, primarily those who have conducted research under the guidance of faculty, should be eligible

## Discussion

This study seeks to examine the role of medical students in the conduct of research on human subjects. Specifically, we used an evidence-based approach to address the eligibility of medical students to serve as PI. Taking into consideration that some aspects of human subject research can challenge even the experts, the question before us is whether a medical student is PI eligible.

Serving as PI is a privilege granted to faculty and other selected professionals associated with an academic institution. Like most other universities, the policy of Alfaisal University do not allow students to serve as PI on human subject research. Several COM students questioned the PI eligibility policy of the institution [2]. 

The results of this study support the current PI eligibility policies at most academic institutions. Most faculty respondents reported that, without proper supervision, medical students do not have the required expertise to lead research projects involving human subjects. Proper guidance limits the potential burden on individuals and the institution and provides the needed learning experience for students who are preparing to conduct research projects [19].

Most of the medical students who responded to the survey reported having no research experience but enough education and training to conduct research studies. Students in this research-based university are highly motivated to conduct research. Hence, most student survey respondents were interested in serving as a PI. Ironically, the majority believed that they were not currently PI eligible. Hence, the opinions of the COM faculty and students on PI eligibility did not differ.

On the other hand, survey respondents promoting student PI eligibility identified university resources available to student investigators. Primarily, members of the IRB occasionally advise medical students as they develop research proposals. Besides, several courses, including biostatistics, epidemiology, and evidence-based medicine, are part of the COM curriculum. The learning objectives of these courses include developing surveys, writing research proposals, learning the art of collecting survey data, performing basic data analysis, and learning the basics of biomedical research ethics [6]. Based on the availability of these institutional resources, the argument was made that with proper training and supervision, a medical student can serve as PI or a Co-PI on IRB exempted or minimal risk studies.

Moreover, to fully engage students in the learning process, they should be encouraged to develop research questions, especially when the question relates to something with which they have personal experience [19]. Student-initiated questions can produce valuable outcomes for the institution and the field of medicine. They keep students motivated and highly engaged in the learning process. The early motivation of clinicians may produce PI eligible physicians who are not only interested but willing to design and direct clinical research projects.

Several important discoveries originated from research projects lead by medical students [20]. For example, in 1922, a Canadian orthopedic surgeon and his medical student discovered insulin, a breakthrough in the practice of medicine that revolutionized the therapy of diabetes. This discovery was a compelling reminder of what can be achieved by well-trained and well-supervised medical students [20].

Another point made by supporters of PI eligibility of medical students was that the NCBE guidelines do not exclude students from the role of PI. Also, physicians take on leadership responsibilities throughout their careers but are not taught how to lead. Serving as a PI is a step in the right direction.

An interesting finding was that significantly more physician faculty were in support of medical students serving as PI than non-physician (PhD, MS) faculty. A physician faculty stated that since medical students are introduced to and trained for clinical practice during college years, we are obligated to train and prepare them at the same time to practice evidence-based medicine during and after graduation. Another faculty noted that obtaining a medical degree should not automatically qualify an inexperienced and unprepared graduate to lead a research project involving human subjects.

The current study was conducted at a private, newly established, nonprofit university. Only faculty, including administrators and students of the COM, were invited to participate. Thus, the primary limitation is the lack of generalizability of study results. The study also suffered from low response rates, especially for medical students. Conducting the study close to the final examination period may have significantly contributed to the low response rates. Furthermore, students were motivated to participate in the study to ensure that their voices are heard. Thus, the low response rate of the medical students may have biased the results towards those who are motivated to take a PI role.

On the other hand, 50% of the COM faculty participated in the study. One of the main reasons was to help in the development of the institutional PI eligibility policy and guidelines. Finally, this study is a step in the right direction for meeting the research challenges with the development of evidence-based PI eligibility policies and procedures.

## Conclusions

The results of this study support the PI eligibility policies at this and other academic institutions of not allowing medical students to serve as PI. Remarkably, the faculty and students had similar a perception of students’ PI eligibility. More physician faculty were in support of medical students serving as a PI than non-physician faculty. The evidence produced in this study will be used in the development of evidence-based PI eligibility policy at this university, and potentially at other universities. The study results are expected to stimulate discussion within the academic community regarding PI eligibility and when PI training should begin.
